# Are We on the Same Page? Action Agenda of the National Conversation on Public Health and Chemical Exposures

**DOI:** 10.1289/ehp.119-a484

**Published:** 2011-11-01

**Authors:** Valerie J. Brown

**Affiliations:** Valerie J. Brown, based in Oregon, has written for *EHP* since 1996. In 2009 she won a Society of Environmental Journalists’ Outstanding Explanatory Reporting award for her writing on epigenetics.


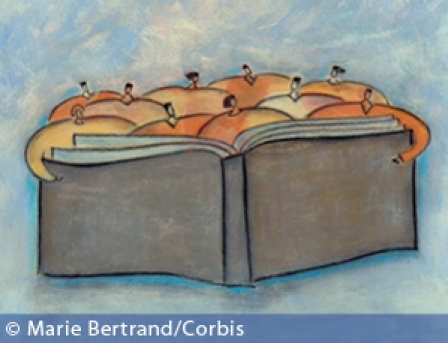
When it comes to the regulation of hazardous chemicals, change in any direction tends to proceed at a snail’s pace. The primary law governing chemicals and health, the Toxic Substances Control Act (TSCA),^1^ has not been revised since it was passed in 1976, due in part to legislative gridlock and lack of consensus among stakeholders.

So it may come as something of a surprise that over the past two years there has been a broad-based, intense, and relatively rancor-free effort to revitalize and rationalize how we manage hazardous industrial and naturally occurring chemicals. The National Conversation on Public Health and Chemical Exposures,^2^ brainchild of former Agency for Toxic Substances & Disease Registry (ATSDR) director Howard Frumkin, delivered its final product—the Action Agenda^3^—on 9 June 2011. “Most of us would agree we don’t want to be around dangerous chemicals. That’s a fairly widely shared value,” Frumkin says. “But you wouldn’t know it based on the amount of polarization and shouting that goes on.”

Now dean of the School of Public Health at the University of Washington, Frumkin became frustrated during his years at the ATSDR, where he concurrently directed the National Center for Environmental Health (NCEH), part of the Centers for Disease Control and Prevention. He wanted to improve federal agency coordination, identify areas of overlapping or redundant responsibility, make information about chemical health effects easily accessible to the general public, streamline and integrate the enormous amount of scientific data on chemicals, and reduce children’s exposure to harmful chemicals. He also wanted to re-establish a strong connection between the public health community and the environmental and occupational health communities. And he sought a fundamental change of perspective from a system based on what the Action Agenda describes as reliance on “treatment after harm has occurred”^3^ to one that prevents health problems.

## Business Unusual

Frumkin took the somewhat daring position that the National Conversation should not operate under the formal protocols of the Federal Advisory Committee Act (FACA),^4^ which governs most federal stakeholder groups. He contracted with RESOLVE, a neutral, nonprofit consensus-building organization, which assembled a voluntary Leadership Council comprising representatives of key governmental players such as the U.S. Environmental Protection Agency (EPA) and the Occupational Safety and Health Administration (OSHA), industry and professional health sciences associations, state health departments, community environmental health organizations, and environmental justice advocates to, he says, “do a deep dive into our national approach.”

Besides the Leadership Council, six special-issue workgroups^5^ each met 10 times. In addition, the National Conversation included web-based discussion sessions and public comment opportunities, as well as 52 public meetings led by local environmental health groups in 24 states. The method used to reach agreement at each level was to ask that participants find principles and recommended actions that they could all “live with” as individuals rather than making decisions on behalf of their organizations or trying to hammer out specific steps to implementation.

The Leadership Council then distilled this wealth of input into the Action Agenda, which lays out seven broad recommendations, each an umbrella for numerous specific goals within what the Action Agenda calls “a comprehensive system that fully protects the public’s health from harmful chemical exposures”^3^:

- protect public health by preventing harmful chemical exposures;
- collect and use information on chemicals and population health to enable effective public health protection;
- achieve a more complete scientific understanding of chemicals and their health effects;
- protect health and wellness in vulnerable communities affected by environmental chemical exposures;
- strengthen the ability of the public to participate effectively in environmental health decision-making.
- strengthen the capacity of the public health and health care provider workforce to address the needs of people exposed to harmful chemicals; and
- reduce harm from chemical emergencies through prevention, planning, and coordination.

## Science in Knots

At the heart of the National Conversation is a mix of old and new research methodologies and policies. TSCA prescribes chemical risk assessments using classic toxicology and exposure scenarios. It requires testing only for newly introduced chemicals, not the tens of thousands in use when the law was enacted. In contrast, the Action Agenda calls for testing of both old and new chemicals and suggests the process could be streamlined by using new analytical tools, including advances in molecular biology, computational toxicology, genomics, proteomics, and bioinformatics.^3p37–39^ The scientific understanding workgroup also recommended developing ways to track total chemical body burden using several different types of tissue samples and assessing potential exposure across both the human lifespan and the chemical life cycle, as well as study of the roles of low-dose, multiple, and cumulative exposures, nonchemical stressors (e.g., psychosocial stress), and genetic predisposition.^6^

Throughout, the Action Agenda calls for including vulnerable and overexposed populations such as children, the elderly, low-income communities, communities of color, tribal communities, and those sensitive to or previously harmed by chemical exposures in the monitoring, testing, and regulation of chemicals in the environment. It notes that with respect to children the toxicological maxim “‘the dose makes the poison’ does not always adequately describe the exposure–health outcome relationship.”^3p23^

To accomplish its ambitious goals, the Action Agenda envisions radically improved communication across sectors and agencies to ensure that officials and the public have full access to the extant scientific and medical knowledge about chemical exposures. For example, most health providers are not well versed in environmental health issues such as pesticide exposure. The Action Agenda calls for integrating environmental health into the education of clinicians and the creation of an educational “pipeline” devoted to increasing the number of professionals from underrepresented communities.^3p63^ The pipeline would include integrating public and environmental issues in undergraduate courses, funding “faculty champions” who would ensure the long-term integration of these issues at universities across the nation, and creating experiential learning opportunities for students from historically marginalized communities.

A related issue is the education of chemical engineers, bioengineers, and materials scientists. Frumkin feels strongly that research and applied scientists need to break out of disciplinary silos. “I maintain that everybody who studies chemistry should learn a bit about human health,” he says. As things stand, he explains, “You can emerge [from your training] oblivious to the fact that the chemicals you’re working with have human health impacts.”

## Back to the Future

The Action Agenda represents a shift toward re-integrating environmental and occupational health with public health. The fields parted company in the 1970s when the EPA and OSHA were established and began to handle environmental monitoring and regulation, says Henry Anderson, state health officer for the Wisconsin Division of Public Health, who co-chaired the Leadership Council. “We wanted to try to reinfuse public health principles into the environmental health programs,” Anderson says.

The first principle of public health is primary prevention—that is, avoiding a harmful exposure or injury altogether rather than treating resulting problems afterward. This is a variant of the precautionary principle^7^ and dovetails with green chemistry and the substitution of alternative chemicals in manufacturing and consumer goods, both of which the Action Agenda endorses.

TSCA’s failure to require toxicity data on most chemicals now in use has hindered the development of potentially less hazardous chemicals, according to the scientific understanding workgroup.^6^ Pam Eliason, industry research program manager at the University of Massachusetts at Lowell’s Toxics Use Reduction Institute, says switching to less hazardous chemical alternatives can be done even in the absence of a clear body of mechanistic studies proving harm from a particular chemical. “If alternatives can be shown to be safer and also technically and economically feasible, then we don’t need to quantify the risk,” she says. Eliason served on the policies and practices workgroup.

## Rubber Hits Road: Regulatory Reform

TSCA’s obsolescence and ineffectiveness elicited a high degree of agreement among many participants. Regulators, industry, and the public are all unhappy with it, says Richard Jackson, a former director of NCEH who chaired the policies and practices workgroup. TSCA reform should give industry “a clear, sensible, transparent regulatory process,” he says. Jackson is now chair of the Environmental Health Sciences Department of the University of California, Los Angeles, School of Public Health.

The Action Agenda’s call for increased chemical testing and reporting requirements was not embraced by all industry stakeholders. Sarah Brozena, senior director for regulatory and technical affairs at the American Chemistry Council, noted during the 2010 public comment period for the Action Agenda that although “[t]he National Conversation’s draft workgroup recommendations represent a good first step in increasing the dialog with the public health community about the important issue of public health and chemical exposures,” some recommendations “reflect a basic lack of understanding of many of the complexities of chemical regulation.”^8^

ACC communications director Scott Jensen says, “ACC was heavily involved in the National Conversation at the beginning of the process, but it quickly became evident that the ‘conversation’ was not being facilitated as a true stakeholder discussion. We preferred to reduce our engagement from the day-to-day working group discussions that, in our view, were not likely to result in constructive policy recommendations. However, we did remain engaged and contributed significantly to the Leadership Council conversations.”

Monsanto’s director of medical sciences and outreach, Daniel Goldstein, sat on the Leadership Council and calls its discussions “very effective and meaningful conversations.” The Leadership Council also included representatives from DuPont and Procter & Gamble.

The primary prevention approach should be attractive to industry because it’s the most cost-effective, Jackson says. And the atmosphere among stakeholders has definitely improved, says Environmental Working Group president Ken Cook, who sat on the Leadership Council. Five years ago there was no agreement that regulatory reform is necessary, he says, whereas now “maybe we’re not on the same page, but we’re reading from the same hymnal. . . . We’ve deepened our understanding of how it is not working on their side and ours.”

## Immovable Forces?

The National Conversation included considerable discussion of ways in which federal agencies could reduce duplicate efforts and waste and minimize turf battles. “You would think it wouldn’t be that hard to come together,” Frumkin says. “But there are lots of protocol barriers—different levels of people who shouldn’t meet because this one is one notch below the administrator and the other is three levels below.”

There are several ways agencies can cooperate, however. For example, says Frumkin, both the ATSDR and the EPA inventory, assemble, and make available the science about the health effects of chemicals. “They’re redundant,” says Frumkin. “Why don’t the two agencies do it just once? They could then devote resources to packaging [the information] in a more user-friendly way. These aren’t hard things to do.”

People working in different sectors such as state public health and environmental agencies and community groups may be able to apply some of the Action Agenda’s principles and actions fairly easily. For example, Anderson is pushing for incorporation of biomonitoring in state public health surveillance programs.

“With all the money going into electronic medical records,” Anderson says, “you ought to be able to capture the information you need to do public health reporting” on chemical exposures and effects. He says adding these data to baseline health information routinely collected by states would make it easier to track population-level effects, compare chemicals with other health factors such as age and nutritional status, and analyze their possible influence on reportable diseases.

## Public Discontent

The public may be the most vexed stakeholder of all. During the comment period at the Washington, DC, meeting launching the project on 26 June 2009, a number of people expressed intense frustration with the ATSDR and other government attempts to address disease clusters and the disproportionate exposure of poor and nonwhite populations to hazardous chemicals.

Similar issues surfaced in community conversations. Renee Hackenmiller-Paradis, program director for environmental health at the Portland-based advocacy group Oregon Environmental Council, conducted a community conversation in Salem, Oregon, where the biggest concerns were pesticides and air and water pollution. “Most people just wanted more information so they could make choices,” Hackenmiller-Paradis says. “The lack of information was very frustrating to everybody.”

The Action Agenda recognizes the legitimacy of these concerns. It suggests creating national data sets accessible through a single website and recommends that the ATSDR “revisit its public health mandate . . . and build community capacity to engage effectively in public decision-making processes.”^3p47^ It also acknowledges that chemical exposures affect people very differently and that individuals with chemical sensitivities or intolerances and those who have previously been harmed by chemical exposures are especially at risk.^3p43^ It further recommends more research on fragrances in consumer products, dust, mold, and mycotoxins indoors.

## The Take-Home

Of course, setting goals is one thing, and shifting entrenched government policies and interest group agendas is something else. Participants are acutely aware of the risk that the Action Agenda will sit on a shelf rather than inform ongoing discourse and action regarding chemical health risks.

“It’s not enough to have an action agenda out there collecting dust,” says Nsedu Obot Witherspoon, executive director of the Children’s Environmental Health Network, a Washington, DC–based national advocacy organization. Obot Witherspoon served as a co-chair of the Leadership Council. Forward movement, Frumkin adds, “will require bold leadership on the part of the agencies.”

Julie Fishman, NCEH/ATSDR associate director for program development, says federal agencies are already talking to each other about the Action Agenda. “The recommendations are being looked at by some longer-standing interagency groups, such as the President’s Task Force on Environmental Health Risks and Safety Risks to Children, the Interagency Working Group on Environmental Justice, and the Interagency Breast Cancer and Environmental Research Coordinating Committee pulled together by the National Institute of Environmental Health Sciences,” Fishman says.

Legislatively, TSCA reform is likely to be the most visible contest over the Action Agenda’s goals. When contacted for this article, an EPA spokeswoman who declined to be named said, “One of EPA’s highest priorities is to make significant and long overdue progress in assuring the safety of chemicals that are used in various products, and found in our environment and our bodies. The Action Agenda contains a number of important recommendations across a range of areas, many of which directly address EPA’s programs and policies.” But the uncertainty surrounding budgets at all levels of government, the weak economic climate, and ideological disagreement over the role of government make action unlikely before the 2012 elections.

Whether or not laws change in the short term, the intent of the National Conversation is resonating throughout the community of interests engaged with chemicals and health and is neatly summarized in the Action Agenda as follows: “Embedded in each recommendation is the fundamental call to make primary prevention the cornerstone of every decision relevant to chemicals and public health so that we can all share in a just and healthy future.”^3p18^
